# First line palliative chemotherapy in elderly patients with advanced soft tissue sarcoma

**DOI:** 10.1186/s13569-015-0026-y

**Published:** 2015-03-24

**Authors:** Nadia Yousaf, Samuel Harris, Juan Martin-Liberal, Susannah Stanway, Mark Linch, Maria Ifijen, Omar Al Muderis, Komel Khabra, Cyril Fisher, Jonathan Noujaim, Ian Judson, Charlotte Benson

**Affiliations:** The Sarcoma Unit, The Royal Marsden Hospital, Fulham Road, London, SW3 6JJ UK

**Keywords:** Sarcoma, Metastatic, Elderly, Chemotherapy

## Abstract

**Background:**

The efficacy and toxicity of first line palliative chemotherapy for soft tissue sarcomas (STS) in the elderly is poorly described.

**Methods:**

Patients over the age of 65 years receiving first line chemotherapy for advanced non-GIST STS January 1998 - January 2012 at the Royal Marsden Hospital were identified. Data regarding survival and predictive factors were collected retrospectively.

**Results:**

120 patients (52 females) with a median age of 72 (range 65–83) were treated. The most common histological subtypes were undifferentiated sarcoma (30%), leiomyosarcoma (27%), angiosarcoma (14%). 42% of patients had high grade tumours. 70% of patients had metastatic disease at presentation; lung metastasis being the most common disease site (72%). 80% received single agent chemotherapy, mostly with doxorubicin (60%). The median number of cycles was 2 (IQR 3). A partial response was reported in 20% of patients with disease stabilisation in a further 20%. 38% of patients were hospitalised for chemotherapy related toxicity. The median overall survival (OS) was 6.5 months (95% CI 4.7-8.3). Anaemia, lymphopenia, hypoalbuminemia, sarcoma subtype and co-morbidities were predictive for overall survival.

**Conclusion:**

The overall survival for elderly patients with STS is poor but several predictive factors have been identified. Hospital admissions for chemotherapy related toxicity are common.

## Background

Soft tissue sarcoma (STS) encompasses a wide spectrum of malignant tumours of mesenchymal origin with variable clinical behaviour. Optimal treatment for those with localised disease is surgery. However, up to half of these patients will later present with metastatic disease. Others present de-novo with locally advanced or metastatic disease which is not amenable to surgical resection. Over 40% of STS are diagnosed in people older than 65 years of age. Patients with inoperable STS have a poor prognosis with a median survival of 12 months or less [1]. The mainstay of treatment for these patients is palliative chemotherapy which has been shown to be beneficial in approximately one half of patients with metastatic STS [1]. Advancing age has been shown to be an independent prognostic factor for poor survival in patients with metastatic STS [1-3]. However, elderly patients are under-represented in clinical trials. A recent analysis of 2636 patient in first line EORTC trials for STS, found only 274 over 65 and 16 over aged over 75 [4]. As such, the efficacy and toxicity of first line palliative chemotherapy for STS in the elderly is poorly described.

We conducted this retrospective study with the aim of describing the efficacy and toxicity associated with first line chemotherapy for advanced STS in an unselected cohort of patients over the age of 65 years treated at a single institution. We aimed to identify predictive prognostic factors.

## Methods

Patients over the age of 65 years diagnosed with STS from January 1998 - January 2012 at the Royal Marsden Hospital were identified via the prospectively maintained sarcoma database.

Only patients with a primary tumour not amenable to surgical resection or those with metastatic disease were included. All patients receiving first line palliative chemotherapy at our institution were included. Patients with gastro intestinal stromal tumour (GIST) were excluded.

Data regarding each patient were retrospectively collected from electronic patient records. Date of diagnosis, age at diagnosis, site of metastasis, chemotherapy agents used, dose reductions, hospital admission during treatment, performance status, serum albumin, sodium, LDH, lymphocyte count, platelet count, date of death or last follow-up were collected. Data on co-morbidities was also collected and the Age Adjusted Charlson Co-morbidity Index (AACCI) [5] for each patient was calculated. However, as locally advanced/metastatic sarcoma was the disease of interest (rather than a co-morbidity) it was excluded from the score. A higher score was indicative of a greater number of co-morbidities. The clinical outcome of each patient was recorded as alive or dead as of 31^st^ of January 2013. Response to treatment was recorded by retrospective review of radiology reports as per Response Evaluation Criteria in Solid Tumors. Patients were re-imaged every 2–3 cycles or earlier if there was clinical suspicion of disease progression.

The study proposal was reviewed and approved by The Royal Marsden Hospital Institutional Audit Committee.

### Statistics

The primary end point was overall survival. The secondary endpoints were response to treatment, admission to hospital secondary to chemotherapy toxicity and exploration of prognostic factors.

Overall survival was measured from the start of chemotherapy to the time of death and censored at last follow-up using the Kaplan-Meier methodology.

The effect of age, histologic subtype, grade, site of metastatic disease, blood parameters and AACCI on response to chemotherapy and hospital admission secondary to chemotherapy toxicity was investigated in univariate analysis by means of the χ^2^ test, Fisher exact test, Mann–Whitney test and Kruskall-Wallis one way ANOVA. Laboratory ranges for blood parameters were as follows; serum albumin (normal range 35 – 50 g/L), sodium (normal range 135 – 145 mmol/L), LDH (normal range 98–192 U/L), lymphocytes (normal range 1.3 – 3.5 × 10^9^/L), platelets (normal range 150 – 400 × 10^9^/L), and haemoglobin (normal range, male 13 – 18 g/dL, female 11.5 – 15.5 g/dl).

A Univariate log-rank analysis was used to investigate these parameters as potential prognostic factors. Multivariate analysis was not performed due to small numbers in subgroups.

## Results

We identified a total for 220 patients ages 65 or over with non-GIST STS between January 1998 to January 2012. 24 patients were excluded as they attended our institution for a second opinion only and 76 patients were excluded as they did not receive chemotherapy. 120 patients (53 (44%) females) with a median age of 72 (range 65–83) were treated and eligible for inclusion in this study.

The most common histological subtypes were undifferentiated sarcoma (30%), leiomyosarcoma (27%), angiosarcoma (14%), liposarcoma (8%), rhabdomyosarcoma (7%) and myofibroblastic tumour (7%). 82 (68%) patients had previous surgical resection with or without radiotherapy for treatment of localised sarcoma. 36 (30%) patients received chemotherapy for locally advanced disease; the remainder had metastatic disease at presentation. Demographic data is shown in Table 1. Lymphopenia at the end of chemotherapy was reversed in only in 2 patients (a patient who progressed after 2 cycles of treatment and another with stable disease). Only 1 patient showed a clinically significant improvement in albumin (but progressed after 2 cycles).

**Table 1:** **Demographic information**

		**Number**	**%**
**Patients**		120	
	Male	68	56.7
	Female	52	43.3
**Median age (range) in years**		73 (65–86)	
**Histology**	Rhabdomyosarcoma	8	6.7
	Angiosarcoma	17	14.2
	Liposarcoma	10	8.3
	Myofibroblastic tumour	8	6.7
	Synovial	2	1.7
	Undifferentiated	36	30
	Leiomyosarcoma	33	27.5
	Other	6	5
**Disease status**	Locally advanced	36	30
	Metastatic	84	70
**Sites of metastatic disease**	Lung	61	
	Liver	15	
	Bone	7	
	Other	14	
**Age Adjusted Charlson Index**	2	7	5.8
	3	17	14.2
	4	4	3.3
	5	3	2.5
	6	5	4.2
	8	24	20
	9	37	30.8
	10	10	8.3
	>10	13	10.8
Anaemia*		65	54.2
**LDH > 250U/L**		71	59.2
**Lymphopenia (<1×10** ^**9**^ **/L)**		34	28.3
**Hypoalbuminemia (<35 g/L)**		48	40

*Haemoglobin < 13 g/L for males, <11.5 g/L females.

### Survival

**Figure 1:**
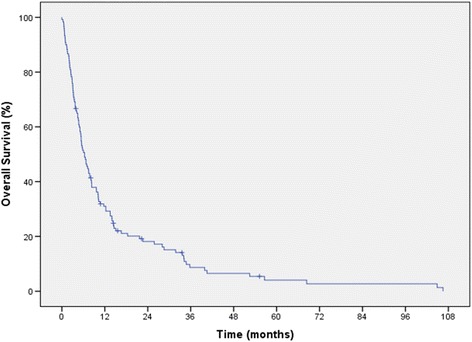
**Kaplan Meier curve for overall survival.**

At the time of analysis 9 patients were still alive. The median overall survival (OS) was 6.5 months (95% CI 4.7-8.3; range 0 – 106) Figure 1. 2 (1.5%) patients died within 30 days of receiving chemotherapy. Univariate analysis revealed lymphocyte count (p = 0.003), albumin (p = 0.003), haemoglobin (p = 0.02), histological subtype (p = 0.02) and AACI (p = 0.03) were predictive of OS (See Table 2).

**Table 2:** **Univariate analysis**

**Variable**	**Groups**	**Median overall survival in months (95% confidence interval)**	**Statistical significance, p**
**Sex**	male	5.4 (3.1 – 7.7)	0.2
	female	6.3 (3.5 – 9.1)	
**Age**	<75 years	6.5 (4.6 – 8.4)	0.3
	>75 years	5.3 (3.9 – 6.6)	
**LDH**	normal	7.1 (4.6 – 9.7)	0.3
	high	5.4 (3.5 – 7.3)	
**Lymphocytes**	normal	7.8 (5.9 – 9.5)	0.003*
	low	3.7 (1.8 - 5.6)	
**Sodium**	normal	5.8 (4.4 – 7.2)	0.8
	low	8.2 (4.2 – 12.3)	
**Albumin**	normal	7.7 (4.9 – 10.5)	0.003*
	low	3.1 (0.9 – 5.1)	
**Haemoglobin**	normal	8.1 (5.7 – 10.5)	0.02*
		5.3 (3.7 – 6.9)	
**Grade**	low	6.0 (4.5 – 7.6)	0.9
	intermediate	7.5 (6.8 – 8.1)	
	high	6.3 (4.8 – 7.9)	
**Histological subtype**	leiomyosarcoma	10.1 (3.9 – 16.4)	0.02*
	angiosarcoma	9.7 (6.4 – 12.9)	
	undifferentiated	3.7 (1.2 – 6.2)	
	other	5.1 (2.9 – 7.2)	
**Sites of visceral involvement**	Lungs	5.7 (4.5 – 7.1)	0.2
	Liver	10.1 (0 – 30.9)	
	bone	6.3 (0.9 – 11.7)	
**Age Adjusted Charlson Index**	<3	9.6 (4.4 – 14.9)	0.03*
	>3	5.6 (3.8 – 7.4)	

*significant factors in univariate analysis. Laboratory ranges for blood parameters were as follows; serum albumin (normal range 35 – 50 g/L), sodium (normal range 135 – 145 mmol/L) lymphocytes (normal range 1 – 3.5 × 10^9^/L), platelets (normal range 150 – 400 × 10^9^/L) , LDH (normal range 100 – 250 U/L) and haemoglobin (normal range, male 13 – 18 g/dL, female 11.5 – 15.5 g/dl).

### Response and toxicity

97 patients (80%) received single agent chemotherapy, most commonly doxorubicin (n = 61) followed by paclitaxel (n = 16). The most common combination chemotherapy regimens were gemcitabine and docetaxel (n = 8) and doxorubicin and ifosfamide (n = 6). The median number of cycles received was 2 (range 1–11). A partial response was reported in 20% of patients, with disease stabilisation in a further 20% (clinical benefit). Univariate analysis did not identify any factors predictive for response to chemotherapy.

38% of patients were hospitalised for chemotherapy related toxicity. Univariate analysis identified only serum albumin as a predictive factor for hospital admission due to toxicity. 32% of patients with a low albumin were admitted to hospital for chemotherapy related toxicity, compared to 24% of patients with a normal serum albumin (p = 0.02).

## Discussion

In this retrospective analysis of real world data from 120 older patients undergoing palliative chemotherapy for STS we have found that the overall survival for this group of patients is poor. Previously reported data from our own institution in patients under the age of 65 showed a median OS of around 1 year [1]. Synovial and liposarcomas represented almost a quarter of this cohort and were found to be independently associated with better survival. In our older cohort, these subtypes accounted for less than 10% of patients and therefore their absence may contribute to the poor survival. We also found that other aspects of tumour histology were an important determinant of prognosis in this elderly population. In particular, median overall survival was significantly better for patients with leiomyosarcomas (10.1 months) and angiosarcomas (9.7) when compared to undifferentiated sarcomas (3.7). This difference was statistically (p = 0.02) and clinically significant and likely reflects differences in the underlying nature of these diseases, and their variation across differing age groups. Undifferentiated sarcoma is often highly aggressive and sometimes resistant to treatment while conversely, the angiosarcoma frequently observed in older patients affects the scalp and face, responds to treatment with paclitaxel and liposomal doxorubicin and may remain relatively localised for many months.

A recent analysis of 274 patients aged over 65 with STS treated with first line chemotherapy within EORTC trials was presented at the 2014 European Society of Medical Oncologists Congress [4]. These patients had a median survival of 9.8 months, but the population differed from ours in that patients were slightly younger (median 68 versus 72 years), with a larger proportion of leiomyosarcomas and were likely to be of a better performance status (more than 85% with a PS of 0–1), since they were patients who had been recruited into clinical trials. Patients with significant co-morbidities are often excluded from clinical trials. We found that more co-morbidity, as assessed by the AACCI was associated with worse OS. This has been reported in other tumour types [6-8] and may be because patients with multiple co-morbidities tolerate chemotherapy less well, or are perhaps sub-optimally treated [9].

Functional capabilities and nutritional status [10] alongside laboratory markers have been shown to predict for survival in older patients [11]. We were unable to gather data regarding the functional ability of patients treated but found a number of laboratory markers to predict for survival. In keeping with previous reports we found that around a quarter of this cohort of patients with advanced STC were lymphopenic and this was associated with worse survival [12]. As the rate of lymphopenia in this elderly population is similar to that previous reported in patients with advanced STS, age related reductions in thymic function are unlikely to be the explanation [13]. Lymphopenia may be an indicator of decreased self-renewal capacity of hematopoietic stem cells thereby contributing to impaired immune function [14] or may also reflect tumour related immunosuppression [15]. Anaemia and hypoalbuminemia in malignancy may be indicative of malnutrition but also reflect a complex tumour related inflammatory cytokine response [16-18]. We collected lymphocyte count and albumin at the end of treatment but found that in the vast majority of patients, even in those that responded to treatment, these markers did not improve. It is difficult to interpret this data but may be related to the overall poor prognosis in this patient group.

We were not able to assess the impact of specific chemotherapy agents on toxicity, response or survival due to the heterogeneity of regimes used. Previous data from our institute in younger patients showed that doublet chemotherapy improved overall survival [1], although this has not been demonstrated in prospective phase 3 studies [19-21]. Only a few single arm studies have specifically investigated tolerability and efficacy of specific chemotherapy regimes in the elderly patients with STS. A small study of daily oral metronomic cyclophosphamide and prednisolone in elderly patients with unresectable STS showed good tolerability and a response rate of almost 27% [22]. Also a retrospective analysis of patients with head and neck angiosarcoma (mainly an elderly population), showed a response rate of greater than 80% and good tolerability [23].

We were unable to identify factors associated with response perhaps because of the smaller number of patients in our study, or perhaps because sarcomas are a heterogonous group of disease with many displaying intrinsic chemotherapy resistance.

We used hospitalisation as a surrogate marker for chemotherapy toxicity and found that over one third of patients were admitted to hospital while undergoing chemotherapy. This is not inconsistent with other data in this age group. A study of patients over the age of 75 undergoing palliative chemotherapy for bowel cancer demonstrated that 42% of patients suffered grade 3 or 4 toxicity [24]. We found that low albumin (possibly due to advanced disease, tumour cachexia or malnutrition) was associated with an increased risk of hospitalisation. Low albumin is known to affect the volume of distribution of chemotherapy drugs and therefore in part there is likely to be a pharmacokinetic explanation for this finding [25].

Comprehensive Geriatric Assessment (CGA) is defined as a multidimensional, interdisciplinary diagnostic process focusing on determining an older person’s co-morbidities, psychosocial, and functional capabilities to develop a coordinated and integrated plan for treatment and long-term follow-up [26]. CGA can detect problems commonly missed in routine clinical assessment [27] in up to 50% of geriatric patients [28] and have been shown to be more sensitive at selecting frail patients than physicians judgement [29]. This could allow for optimisation of co-morbidities prior to assessing older patients for chemotherapy. As age is an independent risk factor for anthracycline induced cardiomyopathy [30] we have adopted an approach of optimising cardiovascular health with cardio-oncology input prior to administration of anthracylines and/or considering liposomal doxorubicin [31].

Despite the potential value, the main limitation to incorporating CGA in oncology clinics is that it requires time and service development. Furthermore, there is a lack of randomised studies to assess the effectiveness of CGA in selecting patient for active oncology treatment versus best supportive care. Attempts have been made to simplify the process of CGA and develop tools incorporating geriatric assessment variables which can be used in clinics. Two large prospective studies [32,33] have demonstrated that scores including geriatric assessment variables have moderate ability to predict for chemotherapy related toxicity and are more sensitive than PS alone [32]. Despite acknowledging the current limitations, The International Society of Geriatric Oncology recommends the use of a CGA to optimise the management of elderly patients with cancer [26].

Our key limitations are those of any retrospective analysis. Data on performance status, weight and specific chemotherapy toxicity were poorly documented and therefore we were unable to assess the effect of these on survival and response.

Older patients with STS have a poor prognosis. Some do benefit from palliative chemotherapy however there is also a high risk of significant toxicity. Selecting appropriate patients to undergo treatment is complex and dependant on several factors including sarcoma subtype, co-morbidities and a number of biomarkers that probably reflect disease burden, such as performance status, low albumin, anaemia etc. These challenges remain, even in an age of targeted therapies as many of these can cause cardiovascular complications [34]. Optimising the management of older people with cancer by developing a robust assessment process and evidence base is imperative. Older patients should be recruited to clinical trials and in some instances specific clinical trials for elderly patients with co-morbidities are warranted.
